# Social Determinants of Antenatal Care Service Use in Ethiopia: Changes Over a 15-Year Span

**DOI:** 10.3389/fpubh.2019.00161

**Published:** 2019-06-25

**Authors:** Seman Kedir Ousman, Ibrahimu Mdala, Viva Combs Thorsen, Johanne Sundby, Jeanette H. Magnus

**Affiliations:** ^1^St. Paul's Hospital Millennium Medical College (SPHMMC), Addis Ababa, Ethiopia; ^2^Faculty of Medicine, University of Oslo, Oslo, Norway; ^3^Department of Global Community Health and Behavioral Sciences, Tulane School of Public Health and Tropical Medicine, New Orleans, LA, United States

**Keywords:** social determinants, ANC utilization, negative binomial RE, Ethiopia, sub-Saharan Africa

## Abstract

**Background:** Improving maternal health in Ethiopia is a major public health challenge. International studies indicate that it is possible to improve maternal health outcomes through action on the Social Determinants of Health (SDH). This study aimed to explore the SDH that influence the antenatal care (ANC) utilization in Ethiopia over time.

**Methods:** The study used data from the nation-wide surveys conducted by the Ethiopian Central Statistical Agency (CSA) and ORC Macro International, USA in 2005, 2011, and 2016. A negative binomial with random effects at cluster level was used to model the number of ANC visits whereas a multilevel binary logistic regression modeled binary responses relating to whether a woman had at least 4 ANC visits or not. The model estimates were obtained with the statistical software Stata SE 15 using the restricted maximum likelihood method.

**Results:** Although the median number of ANC visits significantly increased between 2005 and 2016, the majority of the women do not obtain the four ANC visits during pregnancy as recommended. The odds of having at least four ANC visits were significantly lower among women: below 20 years, those living in the rural areas, having higher birth order, or Muslim. In contrast, higher educational attainment, higher socio-economic status, exposure to mass media, and self-reporting decision empowerment were significantly associated with having at least four ANC visits.

**Conclusion:** The use of ANC visits is driven mostly by the social determinants of health rather than individual health risk. The importance of the various SDHs needs to be recognized by Ministry of Health policy and program managers as a key driving force behind the country's challenges with reaching targets in the health agenda related to maternal health, particularly related to the recommended number of ANC visits.

## Background

The global maternal mortality is at 830 deaths daily, 99% of these are in developing countries (1). Relative to other developing countries, the Ethiopian maternal mortality ratio of 412 per 100,000 live births is high (2). Maternal and newborn deaths can be prevented by high utilization and access to key essential services for mothers including antenatal care (ANC), skilled attendance at birth, and postnatal care (3, 4). “The World Health Organization (WHO) recommended focused ANC (FANC) model consisting of at least four visits for low-risk pregnant women, with targeted interventions at each visit in 2002” (4). Few developing countries, including Ethiopia, have fully embraced and implemented the FANC model. In many resource-limited settings, increasing the number of ANC visits for women with uncomplicated pregnancies beyond four is not associated with improved birth outcomes (5, 6). According to the most recent Ethiopian Demographic Health Survey (EDHS) (2), 62 percent of women who gave birth in the 5 years preceding the 2016 survey had at least one antenatal care visit however, with suboptimal attendance of the recommended visits.

Social determinants are a major underlying cause for inequities in health. International studies suggest that it is possible to improve maternal health outcomes through action on the Social Determinants of Health (SDH), (7–10). This has, however, not been a systematic theme in the health agenda of low and middle-income countries (11, 12). In Ethiopia, the SDH are not systematically addressed in the Health Sector Transformation Plan (HSTP), although it is stated that targeting the social determinants of reproductive health could improve access to quality of services for mothers and children (13). To date, a number of studies have explored individual risk factors of antenatal care utilization in Ethiopia and driven by a complex set of factors that include demographic, cultural, and socio-economic factors, such as age of women, birth order, size of household, education, ethnicity, place of residence, religious background, marital status, employment, income level, and accessibility (14–19). Nonetheless, none of these studies have systematically reviewed the social factors to show their overall pooled effect on the interconnection between social determinants and ANC visit use at national level. Hence, demonstrating of these social factors on ANC use was warranted. The current study will explore the SDH at different levels and its associations with ANC utilization in Ethiopia over a period of 15 years.

## Materials and Methods

The study used data from the three latest EDHSs, conducted by the Ethiopian Central Statistical Agency (CSA) and ORC Macro International, USA, between April 2005—August 2005, December 2010—June 2011, and January 2016—June 2016. The full details of the methods and procedures used in the data collection of each EDHS, are published elsewhere (2, 14, 15). In the current study. We included total of 22, 799 weighted data from: 7306 women collected from 570 Enumeration Areas (EA) (clusters) in 2005; 7908 women from 548 clusters in 2011; and data from 7585 women from 575 clusters in 2016. The eligibility criteria were: being in the reproductive ages 15–49 years, reporting at least one births in the last 5 years preceding the actual survey, and participating in one of the three surveys from any region in the country. The number of interviewed females were 14,070 in the 2005 EDHS, 16,515 in the 2011 EDHS, and 18,500 in the 2016, making a total of 49,085 respondents (2, 14, 15). However, among all female respondents, 22, 799 (46.5%) met the eligibility criteria, and those with complete data on one or more of the variables of interest. Data on these eligible women were pooled from the survey datasets allowing the analysis to span the period 2001 to 2016.

### Outcome Variables

The analyses in the current study were based on two ANC-related outcomes: (1) The total number of ANC visits each participant had in the index pregnancy; (2) A binary outcome based on whether a woman had had four or more visits during the course of the pregnancy or not, according to at that time recommended four visits in the WHO guidelines for FANC (4), as recommended by the Ethiopian Ministry of Health during this period (13).

### Explanatory Variables

Important individual and community level social determinants (SD) were considered in the analyses. *Individual level SD* included: marital status, religion, education level, employment status for both the participant and her partner, empowerment (relating to household decision making and whether the women were involved or not: on her own health care; large household purchases; and visits to family or relatives), household wealth index (low, middle, high), mass media (radio and TV) exposure (no exposure, exposed to either a radio or TV and exposed to both), sex of the household head, maternal age at last birth, birth order. The following *community level SD* were considered: place of residence, urban or rural, and if the region were classified as agrarian, pastoral, or urban.

### Statistical Methods

#### Modeling Number of ANC Visits

The data available contained a significant number of zero counts due to the high number of women not attending ANC at all (71.5% in 2005, and 57.1% in 2011). We addressed these distributional challenges by fitting a negative binomial random effects (NBRE) model to our count data. It is important to note two key study assumptions that should be borne in mind when interpreting our findings: First, given the cross-sectional nature of DHS data, some of the information used in the analysis related to the time of the surveys rather than the time of birth and pregnancy. Secondly, we used 2005 as the reference survey year and estimated the incidence rate ratios (IRR), for 2011 and 2016. Estimates of IRR, which represents the *change* in the number of ANC visits in 2011 compared to 2016, relative to the number of ANC visits in 2005, were obtained from the NBRE model.

#### Modeling Binary Responses

Due to data clustering at the survey level, binary data relating to whether a woman had at least four ANC visits in pregnancy or not, were modeled using a binary logistic multilevel regression model after adjustments for several confounders. We identified the main confounding variables from the literature as: age while giving last birth, order of the last birth, place of residence, and husband's education. Multiple multilevel logistic regression model was used to control the effects of potential confounders and from the model, adjusted odds ratios (AOR) with 95% confidence intervals were obtained. In addition, we computed an estimate of intra-cluster correlation coefficient (ICC), which described the amount of variability in the response variable attributable to differences between the clusters. We then used the McKelvey & Zavoina Pseudo R^2^ to assess the fit of the model (20, 21).

#### Modeling Strategies

Both bivariate (data not given) “see Tables S1a,b.” and adjusted models were fitted to count and binary response data. Individual and cluster level SD that were significantly (*P* ≤ 0.05) associated with having ANC visits were included in the multiple Poisson and logistic regression models while controlling for the effect of other variables contained in the model. The model parameter estimates were obtained in the statistical software StataSE 15 using the restricted maximum likelihood method (REML). The level of significance was set at α = 0.05.

#### Ethical Consideration

The study was conducted by confirming to national and international ethical guidelines for biomedical research involving human subjects (22) including the Helsinki declaration. This study was reviewed and approved from the Regional Committee for Medical and Health Research Ethics (REK) and Norwegian Center for Research data (NSD) at the University of Oslo. Our team also requested permission to have access to the data from the CSA and ICF international by registering online on the website www.dhsprogram.com^1^ and submitting the study protocol (see Additional File 2). We also highlighted the objectives of the study as part of the online registration process. The ORC Macro Inc removed all information that could be used to identify the respondents; hence, confidentiality of the data was maintained.

## Results

### Participants' Characteristics

Out of the 22 799 eligible women, 32.0 % were from the 2005, 34.7% from the 2011 and 33.3% were from the 2016 survey with a mean age of 29.1 (±7) years. As detailed in Table 1, the majority of women were living with their partners (94%), were Christians (65%), residing in rural areas (91%), and from the agrarian regions (92%). Most of the women and their partners were not educated and of low socio-economic status. The employment status of the women improved slightly between 2005 and 2016 (*P* < 0.01), in contrast to the employment status of their partners, which dropped significantly from 97.2% in 2005, 2011 to 79.9% in 2016 (*P* < 0.01). More and more women were involved in at least three major decision making of the household (*P* < 0.01). Over the years, more women had at least one ANC visit (*P* < 0.01).

**Table 1:** Socio-demographic characteristics of female survey participants in the 2005, 2011 and 2016, Ethiopian DHS.

**Background characteristics**	**2005** ***n* (%)**	**2011** ***n* (%)**	**2016** ***n* (%)**	**^**a**^*P*-value**
*n*	7,306	7,908	7585	(Test for trend)
**Individual Level SD**				
**Age when giving last birth (years)**				
<20	992 (13.6)	861 (10.9)	704 (9.3)	<0.01
20-−34	4,308 (59.0)	5,021 (63.5)	4,922 (64.9)	<0.01
35–49	2,006 (27.4)	2,026 (25.6)	1,959 (25.8)	0.03
**Order of the last birth**				
First	1,190 (16.3)	1,399 (17.7)	1,431 (18.9)	<0.01
Second or third	2,089 (28.6)	2,462 (31.1)	2,282 (30.1)	0.05
Fourth or higher	4,027 (55.1)	4,047 (51.2)	3,872 (51.0)	<0.01
**Marital status**				
Not living with partner	535 (7.3)	723 (9.1)	480 (6.3)	0.02
Living with partner	6,771 (92.7)	7,185 (90.9)	7,105 (93.7)	0.02
**Religion**				
^1^Christianity	4,741 (64.9)	5,171 (65.4)	4,600 (60.7)	<0.01
Islam	2,382 (32.6)	2,563 (32.4)	2,824 (37.2)	<0.01
^2^Other	183 (2.5)	169 (2.2)	161 (2.1)	0.12
**Education level of the women**				
No education	5,734 (78.5)	5,270 (66.6)	4,791 (63.2)	<0.01
Primary	1,204 (16.5)	2,270 (28.7)	2,148 (28.3)	<0.01
Secondary and above	368 (5.0)	368 (4.7)	646 (8.5)	<0.01
^b^**Employment status of the women**				
Unemployed	5,497 (75.2)	5,132 (64.9)	5,416 (71.4)	<0.01
Employed	1,808 (24.7)	2,768 (35.0)	2,169 (28.6)	<0.01
**Education level of their partners**				
No education	4,282 (59.2)	3,858 (49.6)	3,346 (47.4)	<0.01
Primary	2,109 (29.2)	3,183 (41.0)	2,731 (38.7)	<0.01
Secondary and above	836 (11.6)	730 (9.4)	985 (13.9)	<0.01
**Employment status of their partners**				
Unemployed	204 (2.8)	148 (1.9)	1,042 (13.7)	<0.01
Employed	7,049 (97.2)	7,649 (97.2)	6,063 (79.9)	<0.01
**In a polygamous relationship**				
No	5,961 (88.6)	6,385 (88.9)	6,343 (89.3)	<0.01
Yes	769 (11.4)	800 (11.1)	762 (10.7)	0.34
**Sex of household head**				
Female	888 (12.2)	1,297 (16.4)	1,115 (14.7)	<0.01
Male	6,418 (87.8)	6,611 (83.6)	6,470 (85.3)	<0.01
^**c**^ **Self-reported empowerment of women**				
Not involved at all in decision making	913 (13.5)	855 (11.9)	789 (11.1)	<0.01
Involved in one	1,306 (19.3)	981 (13.7)	491 (6.9)	<0.01
Involved in two	1,614 (23.8)	1,562 (21.7)	915 (12.9)	<0.01
Involved in at least three	2938 (43.4)	3787 (52.7)	4910 (69.1)	<0.01
**Household wealth index**				
Low	3,074 (42.1)	3,435 (43.4)	3,306 (43.6)	0.06
Middle	1,586 (21.7)	1,628 (20.6)	1,588 (20.9)	0.25
High	2,646 (36.2)	2,845 (36.0)	2,691 (35.5)	0.35
**Exposure to mass media**				
No exposure	4,630 (70.8)	3,214 (47.4)	5,024 (72.6)	<0.01
Exposed to either radio or TV	1,474 (22.6)	1,802 (26.6)	741 (10.7)	<0.01
Exposed to both radio and TV	434 (6.6)	1,767 (26.0)	1,156 (16.7)	<0.01
**Use of antenatal care (ANC)**				
No	5,225 (71.5)	4,517 (57.1)	2,818 (37.2)	<0.01
Yes	2,081 (28.5)	3,391 (42.9)	4,767 (62.8)	<0.01
**Number of ANC Visits**				
0	5,225 (71.5)	4,517 (57.1)	2,818 (37.2)	<0.01
1–3	1,164 (15.9)	1,856 (23.5)	2,342 (30.9)	<0.01
> 3	917 (12.6)	1,535 (19.4)	2,425 (31.9)	<0.01
Median	1.1	1.6	2.4	<0.01
^**d**^ **Frequency of ANC services**				
Inadequate (<4 visits)	6,389 (87.8)	6,373 (80.9)	5,160 (68.2)	<0.01
Adequate (≥ 4 visits)	887 (12.2)	1,508 (19.1)	2,410 (31.8)	<0.01
**Community level SD**				
**Area of residence**				
Urban	632 (8.6)	1,188 (15.0)	965 (12.7)	<0.01
Rural	6,674 (91.4)	6,720 (85.0)	6,620 (87.3)	<0.01
**Region**				
Agrarian	6,691 (91.6)	7,271 (92.0)	6,895 (90.9)	0.13
Pastoralist	448 (6.1)	399 (5.0)	441 (5.8)	0.42
City	167 (2.3)	238 (3.0)	249 (3.3)	<0.01

a Chi-squared test for trend in proportions;

1 Orthodox, Catholic, Protestant

2 Traditional, and other unspecified;

b Total figure may not add to 100 percent due to “do not know” and “missing cases.”

c *Empowerment: decision (i. on her own health care; ii. large household purchases; and iii. visits to family or relatives in the household)*.

d *Frequency: Inadequate by Ethiopian MOH definition less than four ANC visits*.

### Determinants Influencing the Number of ANC Visits

Table 2 presents the estimates of the IRRs and the corresponding 95% confidence intervals (CI) obtained from the NB RE model. Women aged 20–34 years had more ANC visits compared to younger women after controlling for all other variables in the model. Birth order was inversely associated with the number of ANC visits. For example, women whose last births were number four or higher had 46% fewer visits in 2005, 26% fewer visits in 2011 and 15% fewer visits in 2016 than primipara women adjusting for all other variables in the model. Islamic women had across the surveys fewer ANC visits than Christian women. Throughout the survey periods, women in the rural areas had fewer ANC visits than women in the urban areas. Although the attendance improved, women in rural areas had 55% in 2011 and 21% in 2016 fewer ANC visits. Highly educated women (secondary education and above) had 71% more visits in 2005 and 42% more visits in 2011 than illiterate women. However, the effect of education on the number of ANC visits disappeared in 2016. Similar association was also noted related to the partners' education level.

**Table 2:** Social determinants and estimates of negative binomial differences in the number of ANC visits pregnant women attended given as adjusted incident rates ratios (aIRR) using the 2005, 2011, and 2016 Ethiopian Demographic Health Surveys.

**Survey periods**	**2005**		**2011**		**2016**	
**Covariates**	**aIRR (95% CI)**	***P*-value**	**aIRR (95% CI)**	***P*-value**	**aIRR (95% CI)**	***P*-value**
**Individual level SD**						
**Age when giving last birth (ref: <20 years)**						
20–34	1.13 (0.97, 1.33)	0.12	1.21 (1.03, 1.41)	0.02	1.22 (1.05, 1.42)	0.01
35–49	1.14 (0.95, 1.38)	0.16	1.20 (1.00, 1.44)	0.05	1.16 (0.97, 1.38)	0.10
**Order of the last birth (ref: first)**						
Second or third	0.56 (0.49, 0.65)	<0.01	0.88 (0.76, 1.00)	0.06	0.92 (0.82, 1.03)	0.16
Fourth or higher	0.54 (0.46, 0.65)	<0.01	0.74 (0.63, 0.87)	<0.01	0.85 (0.74, 0.98)	0.03
**Religion (ref:christianity**^**1**^**)**						
Islam	1.07 (0.93, 1.23)	0.32	0.83 (0.74, 0.94)	<0.01	0.87 (0.77, 0.97)	0.02
Others^2^	0.49 (0.30, 0.79)	<0.01	0.84 (0.60, 1.18)	0.32	1.16 (0.82, 1.65)	0.40
**Women's education level (ref: no education)**						
Primary	1.36 (1.19, 1.57)	<0.01	1.08 (0.97, 1.20)	0.16	1.09 (0.99, 1.20)	0.07
Secondary and above	1.71 (1.26, 2.32)	<0.01	1.42 (1.04, 1.93)	0.03	0.89 (0.73, 1.07)	0.22
**Woman's employment status (ref: not employed)**						
Employed	1.03 (0.92, 1.16)	0.58	1.25 (1.14, 1.38)	<0.01	1.11 (1.02, 1.21)	0.01
**Partner's education level (ref: no education)**						
Primary	1.00 (0.89, 1.12)	0.99	1.19 (1.08, 1.31)	<0.01	1.13 (1.03, 1.24)	0.01
Secondary and above	1.34 (1.12, 1.61)	<0.01	1.55 (1.27, 1.89)	<0.01	1.06 (0.91, 1.23)	0.43
**Partner's employment status (ref: not employed)**						
Employed	0.74 (0.55, 0.98)	0.03	0.80 (0.56, 1.12)	0.19	1.07 (0.95, 1.20)	0.27
**In a polygamous relationship (ref: no)**						
Yes	1.04 (0.88, 1.23)	0.63	0.93 (0.80, 1.08)	0.36	1.11 (0.97, 1.28)	0.13
**Household wealth index (ref: low)**						
Middle	1.39 (1.21, 1.59)	<0.01	1.23 (1.09, 1.38)	<0.01	1.27 (1.14, 1.41)	<0.01
High	1.84 (1.62, 2.10)	<0.01	1.51 (1.34, 1.70)	<0.01	1.16 (1.05, 1.29)	0.01
**Exposure to media (ref: no mass media exposure)**						
Exposed to either radio or TV	1.21 (1.07, 1.37)	<0.01	1.44 (1.29, 1.61)	<0.01	1.15 (1.01, 1.31)	0.04
Exposed to both radio and TV	1.53 (1.26, 1.86)	<0.01	1.80 (1.61, 2.02)	<0.01	1.14 (1.02, 1.28)	0.02
**Sex of household head (ref: male headed)**						
Female headed	1.13 (0.93, 1.37)	0.21	0.91 (0.78, 1.05)	0.20	0.96 (0.84, 1.09)	0.51
**Self-reported empowerment of women (ref: not involved at all in decision making)**						
Involved in one	0.78 (0.65, 0.93)	0.01	1.74 (1.44, 2.10)	<0.01	1.54 (1.27, 1.87)	<0.01
Involved in two	1.03 (0.86, 1.22)	0.78	1.77 (1.49, 2.10)	<0.01	1.53 (1.29, 1.81)	<0.01
Involved in at least three	1.05 (0.89, 1.24)	0.54	2.10 (1.79, 2.45)	<0.01	1.49 (1.30, 1.71)	<0.01
**Community level SD**						
**Area of residence (ref: urban)**						
Rural	0.38 (0.29, 0.49)	<0.01	0.45 (0.37, 0.55)	<0.01	0.79 (0.65, 0.96)	0.02
**Contextual region (ref:agrarian)**						
Pastoralist	1.37 (1.08, 1.72)	0.01	1.08 (0.86, 1.36)	0.49	1.01 (0.82, 1.23)	0.93
City	3.39 (2.15, 5.33)	<0.01	2.17 (1.55, 3.06)	<0.01	1.19 (0.83, 1.70)	0.33

1 *Orthodox, Catholic, Protestant*,

2 *Traditional, and other unspecified; ref, reference category; aIRR, adjusted incidence rate ratios*.

*a:adjusted for: age at last birth, order of last birth, religion, place of residence, region, women education, women employment, husband education, husband employment, polygams relation, wealth, media, sex of household head, empowerment*.

**Figure 1:**
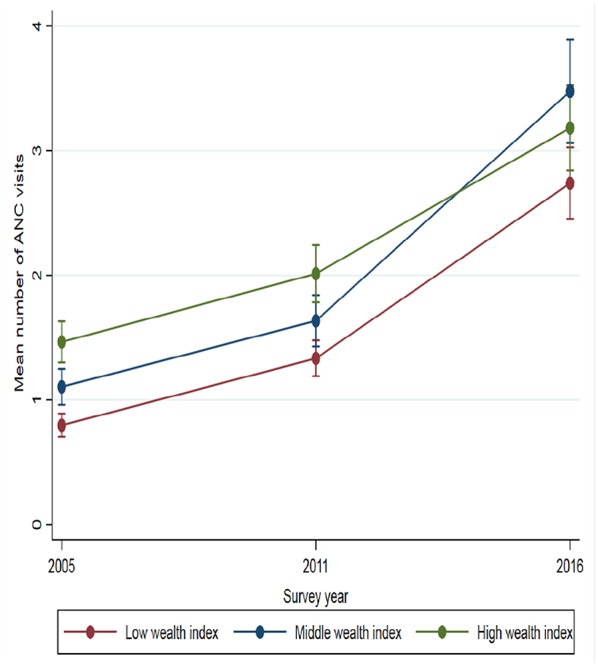
Mean distribution of ANC visits by household wealth index at each survey year.

The analyses (Table 2) also exposed that women with jobs had 25% more ANC visits in 2011 and 11% more ANC visits in 2016 than those unemployed. Household wealth index was significantly associated with the number of ANC visits in all three-survey years. Women from households with middle wealth indices had 39% in 2005, 23% in 2011 and 27% in 2016 more visits than women from low wealth indexed households. Women from high indexed households had 84% in 2005, 51% in 2011, and 16% in 2016 more ANC visits than women from households with low wealth index. In all three surveys, women exposed to mass media had more ANC visits than women who were not exposed to any mass media. For instance, being exposed to both radio and TV increased the number of ANC visits by 53% in 2005, 80% in 2011, and 14% in 2016. Similarly, women empowerment was also found to be an important determinant for ANC use. Women who were empowered stating three or more major household decisions had 2.1 times more in 2011 and 49% higher ANC visits in 2016 than women who were not empowered at all controlling for all other variables in the model. Figure 1 show selected social determinant for the evolution of inequity in the mean distribution of ANC visits.

### Trends and Changes in the Number of ANC Visits in Ethiopia

The changes in the number of ANC visits in each category of the predictors with the 2005 survey as the reference was observed (not shown) “see Table S2” The number of ANC visits increased in each category from 2005 to 2016. For instance, women below 20 years had 43% more ANC visits in 2011 and 2.7 times more ANC visits in 2016 relative to the number of antenatal visits in 2005. Illiterate women had 60% more visits in 2011 and 3.08 times more visits in 2016, relative to the number of visits they had in 2005. Rural women had 52% more visits in 2011 than in 2005 and 3.09 times more visits in 2016 than in 2005. Similar trends were also observed in different categories of employment status, partner's level of education and their employment status, levels of household wealth index, exposure to mass media and the levels of empowerment of women as measured by self-reported participation in decision making.

### Changes in Completing Four or More ANC Visits Over Time

Changes in having at least four ANC visits during any pregnancy in each key social determinants over time were observed after controlling for potential confounding effects of age while giving last birth, order of the last birth, place of residence, and husband's education. Between 2011 and 2016, the odds of ANC use among pregnant women increased significantly by 2-fold: 1.13 (95% CI: 0.96 - 1.32, *p* = 0.13) to 2.14 (95% CI: 1.84 - 2.49, *p* < 0.01) (Table 3). Furthermore, the results for the covariates included in the multilevel logistic regression model as controls (not shown) “see Table S3” conformed that mothers age, birth order of the child, religion, place of residence, women's education, wealth index, media exposure, sex of household head, and women empowerment were significant determinants for completing four or more ANC visits. Overall for these Ethiopian women, the odds of having at least four ANC visits were significantly higher in 2016 than in 2005 (*P* < 0.01). We obtained an intra-cluster correlation coefficient (ICC) of 0.11 from the adjusted multilevel logistic regression. This means that the differences between the clusters account for 11% of the variability in the distribution of women with adequate ANC visits. Based on the McKelvey & Zavoina Pseudo R^2^, the models provided a good fit of the data.

**Table 3:** Adjusted multiple multilevel logistic regression model showing the association between selected individual and social covariates of women reporting at least four ANC visits in the 2011 and 2016 surveys relative to the 2005 Ethiopian Demographic Health Survey.

**Survey periods**	**2011**		**2016**	
**Covariates**	**AOR (95% CI)**	***P*-value**	**AOR (95% CI)**	***P*-value**
**Overall time effect (ref: 2005)**	**1.13 (0.96, 1.32)**	**0.13**	**2.14 (1.84, 2.49)**	**<0.01**
**Individual level SD**				
**Age when giving last birth** (years)				
<20	0.64 (0.41, 0.99)	0.05	1.36 (0.91, 2.04)	0.14
20–34	1.18 (0.97, 1.43)	0.10	2.32 (1.93, 2.79)	<0.01
35–49	1.32 (0.98, 1.79)	0.07	2.16 (1.62, 2.88)	<0.01
**Order of the last birth**				
First	0.83 (0.60, 1.15)	0.27	1.19 (0.88, 1.62)	0.27
Second or third	1.19 (0.97, 1.47)	0.10	2.53 (2.07, 3.08)	<0.01
Fourth or higher	1.29 (0.96, 1.75)	0.10	2.23 (1.79, 3.19)	<0.01
**Religion**				
Christianity^1^	1.23 (1.01, 1.51)	0.04	2.24 (1.84, 2.72)	<0.01
Islam	0.98 (0.76, 1.26)	0.86	1.97 (1.55, 2.51)	<0.01
Others^2^	1.43 (0.36, 5.65)	0.61	2.41 (0.65, 8.93)	0.19
**Women's education level**				
No education	1.20 (0.99, 1.47)	0.07	2.63 (2.18, 3.17)	<0.01
Primary	1.12 (0.83, 1.51)	0.45	2.01 (1.51, 2.67)	<0.01
Secondary and above	0.73 (0.43, 1.24)	0.25	0.78 (0.50, 1.22)	0.28
**Woman's employment status**				
Not employed	1.09 (0.89, 1.33)	0.42	2.31 (1.92, 2.79)	<0.01
Employed	1.15 (0.90, 1.47)	0.28	1.90 (1.49, 2.42)	<0.01
**Partner's education level**				
No education	1.08 (0.85, 1.37)	0.51	2.67 (2.15, 3.32)	<0.01
Primary	1.46 (1.12, 1.90)	<0.01	2.58 (2.00, 3.33)	<0.01
Secondary and above	0.80 (0.56, 1.14)	0.21	1.05 (0.77, 1.42)	0.77
**Partner's employment status**				
Not employed	0.89 (0.47, 1.69)	0.72	1.55 (0.97, 2.50)	0.07
Employed	1.15 (0.98, 1.35)	0.09	2.21 (1.89, 2.59)	<0.01
**In a polygamous relationship**				
No	1.15 (0.98, 1.36)	0.09	2.19 (1.86, 2.56)	<0.01
Yes	0.95 (0.60, 1.49)	0.82	1.82 (1.20, 2.76)	0.01
**Household wealth index**				
Low	1.38 (1.02, 1.88)	0.04	3.41 (2.57, 4.52)	<0.01
Middle	1.41 (0.96, 2.09)	0.08	3.27 (2.28, 4.70)	<0.01
High	0.97 (0.79, 1.20)	0.81	1.41 (1.15, 1.73)	<0.01
**Exposure to media**				
No mass media exposure	1.08 (0.86, 1.36)	0.49	2.47 (2.03, 3.00)	<0.01
Exposed to either radio or TV	1.01 (0.76, 1.35)	0.94	2.36 (1.74, 3.20)	<0.01
Exposed to both radio and TV	1.10 (0.79, 1.53)	0.59	1.40 (1.00, 1.96)	0.05
**Sex of household head**				
Male headed	1.25 (1.05, 1.48)	0.01	2.29 (1.95, 2.70)	<0.01
Female headed	0.57 (0.38, 0.86)	0.01	1.30 (0.89, 1.90)	0.17
**Self-reported empowerment of women**				
Not involved at all in decision making	0.71 (0.44, 1.15)	0.16	2.02 (1.31, 3.11)	<0.01
Involved in one	1.19 (0.81, 1.76)	0.38	3.69 (2.49, 5.48)	<0.01
Involved in two	0.97 (0.71, 1.33)	0.84	2.09 (1.51, 2.87)	<0.01
Involved in at least three	1.27 (1.03, 1.58)	0.03	2.03 (1.66, 2.47)	<0.01
**Community level SD**				
**Area of residence**				
Urban	0.71 (0.52, 0.98)	0.04	0.76 (0.56, 1.04	0.09
Rural	1.29 (1.06, 1.56)	0.01	3.00 (2.50, 3.58)	<0.01
**Contextual region**				
Agrarian	1.37 (1.10, 1.71)	0.01	2.87 (2.34, 3.52)	<0.01
Pastoralist	0.74 (0.54, 1.00)	0.05	1.34 (1.00, 1.80)	0.05
City	1.06 (0.73, 1.53)	0.77	1.63 (1.13, 2.35)	0.01

1 *Orthodox, Catholic, Protestant*.

2 *Traditional, and other unspecified; AOR, adjusted odds ratios; ref, reference category*.

*a: adjusted for: time effect, age at last birth, order of lastbirth, religion, place of residence, region, women education, women employment, husband education, husband employment, polygams relation, wealth, media, sex of household head, empowerment*.

## Discussion

This is the first Ethiopian study exploring the association between various SDH and the use of ANC during pregnancy over a longer time span. The study demonstrates that the ANC use in Ethiopia increased significantly from 2005 (28.5%) to 62.8 in 2016. However, only 32% of the Ethiopian women met the current Ethiopian policy recommendation of four ANC visits (Table 1). This is somewhat lower than in studies from other sub–Saharan African countries (41% to 87%) (23–25). The finding indicated that ANC use depended on the joint effect of individual and community level determinants (Table 1). We explored, on the basis of available evidences, some of those factors which act as social determinants of ANC use.

Globally, economically disadvantaged women suffer from maternal health inequity facilitated by several identifiable and modifiable social determinants, including household wealth (26–28). We demonstrate in this study that inequity is still there and the increase in utilization included the most vulnerable women, with low economic status or no formal education. Fortunately, the magnitude of disparity detected in our study was smaller than earlier studies in Ethiopia and other developing countries (29–35). Likewise, it is noteworthy that having a partner with a high educational level was one of the social determinants for ANC attendance in Ethiopia. The role of men in ANC use in a patriarchal society like Ethiopia, where women might seek a husband's permission or approval before taking decisions related to care, warrants further studies. The importance of men's education on maternal health issues, as well as the use of ANC, may play a critical role when politically shaping family priorities and health-seeking behaviors.

The current study demonstrates that there is significant differences in the use of ANC services between women of different socio-demographic, cultural, and geographic backgrounds. Women from rural communities have had fewer ANC visits, with significant variations in the number of ANC visits across administrative regions. This should guide regional and local initiatives aimed at increasing utilization of ANC and other preventive services. Especially, pregnant women from pastoralist regions might require special support as poor health resources might be attenuated by a lower literacy rate in this population (36). Muslim women had fewer ANC visits than Christian women across all survey years. This warrants exploration and more in-depth qualitative studies.

Our findings also indicate that women, not empowered in household decision-making or exposed to any form of mass media, have lower ANC utilization. This finding is consistent with other studies (37–40). For the last two decades the political environment in Ethiopia has enabled more options for accessing public health information (41). Findings from our logistic regression model also suggest that the odds of having at least four ANC visits in pregnancy were significantly higher among women in higher age groups and those with higher education status. In general, Ethiopian health policy initiatives such as deploying Health Extension Workers (HEWs) and Women Developmental Armies (WDA), thus identify pregnant woman in rural communities earlier, might have contributed to increased awareness through health promotion, aimed at improved ANC utilization among poor women living in rural areas (42).

### Strength and Weakness of the Study

The findings from this study are based on three waves of survey data that were collected by two reputable institutions: The Ethiopian Central Statistical Agency and the ORC Macro International, USA. The sample sizes of the three surveys were large providing high statistical power. The utilization of count data modeling provides methodologically advantage by taking discrete observations (counts) into account that made the model estimates reliable. Although these data were collected at three different time points, the outcome measures and the predictors were taken from different women in each survey. This makes it impossible to relate the changes in the utilization of the ANC services to the individual level but offer good estimates at the community or village level. Also, the cross-sectional nature of the data does not allow to draw causal inferences and DHS data are associated with recall bias that data was collected retrospectively on events that took place 5 years before the surveys.

## Conclusion

The maternal health status could not be improved without fundamental changes in education, household wealth status, employment, media exposure, and empowerment. The importance of the various SDH needs to be recognized by the Ministry of Health policy and program managers as a key driving force behind the country's challenges with reaching targets in the health agenda related to maternal health, particularly related to the recommended number of ANC visits. To ensure adequate use of antenatal service in Ethiopia, upstream approaches that address social issues need to be considered. Such efforts could help improve health equity for maternal health outcomes in the country. More research should investigate whether the SDH identified in this study impact other maternal health indicators.

## Ethics Statement

The EDHS data used in this study has an ethical clearance from at least one of the following institutes: Ethiopian Central Statistical Authority (CSA); Federal Ministry of Health; National Research Ethics Review Committee (NRERC); and the Institutional Review Board of ICF International through DHS Program. Consent was obtained from each study participant before conducting an interview. We obtained the data by submitting the study protocol through registering online on the website www.dhsprogram.com.

## Author Contributions

SO, VT, and JM conceived the research. IM and SO designed the study, analyzed the data, and developed the first draft. JM, JS, IM, and VT critically reviewed and edited the manuscript for intellectual content. All authors revised the final document, read and approved the final manuscript.

### Conflict of Interest Statement

The authors declare that the research was conducted in the absence of any commercial or financial relationships that could be construed as a potential conflict of interest.

## Footnotes

^1^http://dhsprogram.com/data/available-datasets.cfm

## Abbreviations

ANC: Antenatal care
AOR: Adjusted Odds Ratio
CSA: Central Statistical Agency
EA: Enumeration Areas
EDHS: Ethiopian Demographic Health Survey
FANC: Focused ANC
HEP: Health Extension Workers
HSTP: Health Sector Transformation Plan
ICC: Intra-Cluster Correlation Coefficient
IRR: Incidence Rate Ratio
NBRE: Negative Binomial Random Effects
NSD: Norwegian Center for Research data
REK: Regional Committee for Medical and Health Research Ethics
REML: Restricted Maximum Likelihood
SDH: Social Determinants of Health
USA: United States of America
WDA: Women Developmental Armies
WHO: World Health Organization.
