# Supplementary material for: Social Determinants of Antenatal Care Service Use in Ethiopia: Changes Over a 15-Year Span
Source: Front Public Health. 2019 Jun 25;7:161. doi: 10.3389/fpubh.2019.00161 (PMC6603173; doi:10.3389/fpubh.2019.00161)
Supplement: Supplementary file 1 [file Table_1.docx]

**Additional file Table S1a.** Estimates of negative binomial for differences in the number of ANC visits attended by selected predictors given as unadjusted incident rates ratios (uIRR) in the 2005, 2011 and 2016 Ethiopian DHS

| **Parameters** | **2005** | P-value | **2011** | P-value | **2016** | P-value |
| --- | --- | --- | --- | --- | --- | --- |
| Covariates | Unadjusted Model | P-value | Unadjusted Model | P-value | Unadjusted Model | P-value |
| Covariates | uIRR (95 % CI) | P-value | uIRR (95 % CI) | P-value | uIRR (95 % CI) | P-value |
| **Survey Year** (ref: 2005 ) | 1 (1,1) |  | 1.65 (1.54, 1.77) | 0.000*** | 2.66 (2.48, 2.85) | 0.000*** |
| **Individual level SD** |  |  |  |  |  |  |
| **Age when giving last birth** (ref: < 20 years) |  |  |  |  |  |  |
| 20 – 34 | 1.01 (0.84, 1.20) | 0.940 | 1.00 (0.88, 1.15) | 0.945 | 0.96 (0.88, 1.05) | 0.368 |
| 35 – 49 | 0.91 (0.74, 1.11) | 0.332 | 0.87 (0.75, 1.01) | 0.066* | 0.76 (0.69, 0.84) | 0.000*** |
| **Order of the last birth** (ref: First) |  |  |  |  |  |  |
| Second or third | 0.79 (0.67, 0.93) | 0.005*** | 0.82 (0.74, 0.91) | 0.000*** | 0.84 (0.78, 0.89) | 0.000*** |
| Fourth or higher | 0.71 (0.59, 0.85) | 0.000*** | 0.66 (0.58, 0.74) | 0.000*** | 0.69 (0.64, 0.75) | 0.000*** |
| **Religion (ref:** Christianity^1^) |  |  |  |  |  |  |
| Islam | 0.67 (0.53, 0.84) | 0.000*** | 0.81 (0.72, 0.91) | 0.000*** | 0.88 (0.80, 0.97) | 0.010** |
| Others^2^ | 0.40 (0.20, 0.81) | 0.011** | 0.60 (0.44, 0.82) | 0.002*** | 1.22 (0.95, 1.58) | 0.118 |
| **Marital status** (ref: Not living with partner |  |  |  |  |  |  |
| Living with partner | 1.11 (0.89, 1.39) | 0.359 | 1.28 (1.11, 1.47) | 0.000*** | 1.02 (0.92, 1.14) | 0.660 |
| **Women’s Educational level (**ref: No Education) |  |  |  |  |  |  |
| Primary Education | 1.55 (1.32, 1.83) | 0.000*** | 1.52 (1.38, 1.66) | 0.000*** | 1.32 (1.24, 1.40) | 0.000*** |
| Secondary Education and Higher | 3.78 (2.88, 4.97) | 0.000*** | 2.66 (2.22, 3.19) | 0.000*** | 1.69 (1.54, 1.86) | 0.000*** |
| **Employment status of woman (ref:** Not-Employed) |  |  |  |  |  |  |
| Employed | 1.04 (0.90, 1.20) | 0.570 | 1.26 (1.15, 1.37) | 0.000*** | 1.08 (1.02, 1.14) | 0.007*** |
| **Husband’s Educational level (**ref: No Education) |  |  |  |  |  |  |
| Primary Education | 1.36 (1.18, 1.56) | 0.000*** | 1.35 (1.24, 1.48) | 0.000*** | 1.27 (1.20, 1.35) | 0.000*** |
| Secondary Education and Higher | 2.71 (2.21, 3.34) | 0.000*** | 2.30 (1.99, 2.67) | 0.000*** | 1.65 (1.51, 1.79) | 0.000*** |
| **Employment status of partner** (ref: Not-Employed) |  |  |  |  |  |  |
| Employed | 0.58 (0.42, 0.79) | 0.001*** | 0.85 (0.64, 1.13) | 0.261 | 1.08 (1.00, 1.16) | 0.057* |
| **In a polygamous relationship** (ref: No) |  |  |  |  |  |  |
| Yes | 0.91 (0.73, 1.14) | 0.420 | 0.85 (0.73, 0.98) | 0.025** | 0.99 (0.90, 1.08) | 0.780 |
| **Woman’s empowerment** (ref: Not empowered |  |  |  |  |  |  |
| Empowered | 1.13 (0.87, 1.48) | 0.343 | 1.90 (1.60, 2.26) | 0.000*** | 1.62 (1.46, 1.79) | 0.000*** |
| **Household wealth index** (ref: Low) |  |  |  |  |  |  |
| Middle | 1.38 (1.16, 1.64) | 0.000*** | 1.28 (1.14, 1.43) | 0.000*** | 1.22 (1.13, 1.31) | 0.000*** |
| High | 2.49 (2.10, 2.95) | 0.000*** | 2.14 (1.91, 2.40) | 0.000*** | 1.51 (1.41, 1.62) | 0.000*** |
| **Exposure to Media** (ref: no mass media exposure) |  |  |  |  |  |  |
| Exposed to either radio or TV | 1.56 (1.31, 1.85) | 0.000*** | 1.56 (1.39, 1.75) | 0.000*** | 1.21 (1.11, 1.32) | 0.000*** |
| Exposed to Both radio and TV | 2.34 (1.78, 3.08) | 0.000*** | 2.10 (1.87, 2.36) | 0.000*** | 1.45 (1.34, 1.57) | 0.000*** |
| **Sex of household head** (ref: Male headed) |  |  |  |  |  |  |
| Female headed | 0.99 (0.83, 1.18) | 0.031** | 0.94 (0.84, 1.04) | 0.245 | 0.96 (0.89, 1.03) | 0.000*** |
| **Empowerment of women** (ref: Not involved at all) |  |  |  |  |  |  |
| Involved in one | 0.76 (0.59, 0.96) | 0.023** | 1.81 (1.51, 2.16) | 0.000*** | 1.48 (1.30, 1.69) | 0.000*** |
| Involved in two | 1.07 (0.85, 1.36) | 0.554 | 1.76 (1.50, 2.08) | 0.000*** | 1.55 (1.38, 1.73) | 0.000*** |
| Involved in at least three | 1.21 (0.97, 1.52) | 0.091* | 2.21 (1.89, 2.57) | 0.000*** | 1.63 (1.48, 1.79) | 0.000*** |
| **Community level SD** |  |  |  |  |  |  |
| **Area of Residence (ref: urban )** |  |  |  |  |  |  |
| Rural | 0.08 (0.06, 0.12) | 0.000*** | 0.21 (0.17, 0.26) | 0.000*** | 0.40 (0.35, 0.46) | 0.000*** |
| **Contextual Region** (ref:Agrarian) |  |  |  |  |  |  |
| Pastoralist | 0.89 (0.58, 1.37) | 0.591 | 0.84 (0.64, 1.09) | 0.190 | 0.71 (0.60, 0.84) | 0.000*** |
| City | 12.00 (7.25, 19.88) | 0.000*** | 5..11 (3.78, 6.91) | 0.000*** | 2.36 (1.95, 2.86) | 0.000*** |

*sig. at 10% level; **sig. at 5% level; ***sig. at 1% level; ^1^Orthodox, Catholic, Protestant ^2^Traditional, and other unspecified; ref: reference category

uIRR: Unadjusted incidence rate ratios

**Additional file Table S1b.** Bivariate analyses of logistic regression model showing social factors associated with having at least four ANC visits by survey year-2005, 2011, and 2016 Ethiopia DHS

| **Parameters** | **2005** | P-value | **2011** | P-value | **2016** | P-value |
| --- | --- | --- | --- | --- | --- | --- |
| Covariates | COR (95 % CI) | P-value | COR (95 % CI) | P-value | COR (95 % CI) | P-value |
| **Overall time effect** (ref: 2005) | 1 (1,1) |  | 1.23 (1.11, 1.37) | 0.000*** | 1.82 (1.64, 2.01) | 0.000*** |
| **Individual level SD** |  |  |  |  |  |  |
| **Age when giving last birth** (years) | 1 (1,1) |  | 1 (1,1) |  | 1 (1,1) |  |
| 20 – 34 | 1.22 (0.91, 1.64) | 0.182 | 1.53 (1.15, 2.03) | 0.004*** | 1.57 (1.23, 2.01) | 0.000*** |
| 35 – 49 | 0.91 (0.65, 1.28) | 0.601 | 1.41 (1.03, 1.94) | 0.032** | 1.23(0.93, 1.61) | 0.144 |
| **Order of the last birth**  (ref: First) | 1 (1,1) |  | 1 (1,1) |  | 1 (1,1) |  |
| Second or third | 0.70 (0.55, 0.88) | 0.002*** | 0.73 (0.60, 0.88) | 0.001*** | 0.78 (0.66, 0.92) | 0.004*** |
| Fourth or higher | 0.43 (0.32, 0.59) | 0.000*** | 0.61 (0.47, 0.78) | 0.000*** | 0.46 (0.37, 0.57) | 0.000*** |
| **Religion (ref:** Christianity^1^) | 1 (1,1) |  | 1 (1,1) |  | 1 (1,1) |  |
| Islam | 0.62 (0.45, 0.84) | 0.003*** | 0.73 (0.59, 0.91) | 0.005*** | 0.55 (0.44, 0.68) | 0.000*** |
| Others^2^ | 0.24 (0.06, 0.96) | 0.044** | 0.44 (0.18, 1.06) | 0.066* | 0.85 (0.40, 1.81) | 0.672 |
| **Women's marital status** (Not: living with partner) | 1 (1,1) |  | 1 (1,1) |  | 1 (1,1) |  |
| Living with partner | 1.49 (1.06, 2.08) | 0.022** | 1.24 (0.95, 1.61) | 0.114 | 1.14 (0.89,1.48) | 0.301 |
| **Women’s Educational level (**ref: No Education) | 1 (1,1) |  | 1 (1,1) |  | 1 (1,1) |  |
| Primary Education | 2.74 (2.13, 3.52) | 0.000*** | 2.31 (1.91, 2.78) | 0.000*** | 1.95 (1.66, 2.30) | 0.000*** |
| Secondary Education & Above | 12.89 (9.27, 17.92) | 0.000*** | 8.00 (5.92, 10.80) | 0.000*** | 4.25 (3.42, 5.27) | 0.000*** |
| **Employment status of woman (ref:** Not-working) | 1 (1,1) |  | 1 (1,1) |  | 1 (1,1) |  |
| Working | 1.14 (0.92, 1.42) | 0.224 | 1.26 (1.06, 1.50) | 0.009*** | 1.07 (0.92, 1.24) | 0.395 |
| **Husband’s Educational level (**ref: No Education) | 1 (1,1) |  | 1 (1,1) |  | 1 (1,1) |  |
| Primary Education | 1.88 (1.45, 2.45) | 0.000*** | 2.01 (1.64, 2.45) | 0.000*** | 1.68 (1.41, 2.01) | 0.000*** |
| Secondary Education & Above | 7.63 (5.70, 10.20) | 0.000*** | 4.92 (3.80, 6.38) | 0.000*** | 3.50 (2.84, 4.31) | 0.000*** |
| **Employment status of partner (ref:** Not-working) | 1 (1,1) |  | 1 (1,1) |  | 1 (1,1) |  |
| Working | 0.98 (0.67, 1.44) | 0.938 | 0.80 (0.52, 1.24) | 0.316 | 1.26 (1.03, 1.53) | 0.023** |
| **In a polygamous relationship** | 1 (1,1) |  | 1 (1,1) |  | 1 (1,1) |  |
| Yes | 0.75 (0.52, 1.08) | 0.121 | 0.72 (0.53, 0.98) | 0.037** | 0.70 (0.55, 0.90) | 0.005*** |
| **Household Wealth Index** (ref: Low) | 1 (1,1) |  | 1 (1,1) |  | 1 (1,1) |  |
| Middle | 1.81 (1.22, 2.68) | 0.003*** | 1.50 (1.12, 2.01) | 0.007*** | 1.68 (1.34, 2.11) | 0.000*** |
| High | 7.50 (5.38, 10.47) | 0.000*** | 4.87 (3.79, 6.26) | 0.000*** | 4.29 (3.55, 5.17) | 0.000*** |
| **Exposure to Media** (ref: no mass media exposure) | 1 (1,1) |  | 1 (1,1) |  | 1 (1,1) |  |
| Exposed to either radio or TV | 2.08 (1.57, 2.75) | 0.000*** | 1.54 (1.21, 1.97) | 0.001*** | 2.13 (1.70, 2.67) | 0.000*** |
| Exposed to Both radio and TV | 3.87 (2.63, 5.69) | 0.000*** | 3.21 (2,53, 4.06) | 0.000*** | 2.69 (2.23, 3.25) | 0.000*** |
| **Sex of household head** (ref: Male headed) |  |  |  |  |  |  |
| Female headed | 1.25 (0.98, 1.60) | 0.072* | 0.90 (0.74, 1.10) | 0.318 | 0.98 (0.82, 1.16) | 0.031** |
| **Women’s empowerment** (ref: Not involved in any of the decisions | 1 (1,1) |  | 1 (1,1) |  | 1 (1,1) |  |
| Involved in one decision | 0.80 (0.51, 1.25) | 0.325 | 1.42 (0.94, 2.13) | 0.093* | 1.77 (1.23, 2.54) | 0.002*** |
| Involved in two of the decisions | 1.22 (0.81, 1.86) | 0.336 | 1.40 (0.96, 2.05) | 0.080* | 1.40 (1.01, 1.95) | 0.043** |
| Involved in three of the decisions | 1.37 (0.93, 2.04) | 0.115 | 2.26 (1.60, 3.21) | 0.000*** | 1.61 (1.22, 2.12) | 0.001*** |
| **Community level SD** |  |  |  |  |  |  |
| **Area of Residence** (ref: urban ) | 1 (1,1) |  | 1 (1,1) |  | 1 (1,1) |  |
| Rural | 0.02 (0.01, 0.03) | 0.000*** | 0.05 (0.03, 0.06) | 0.000*** | 0.12 (0.10, 0.16) | 0.000*** |
| **Contextual Region** (ref:Agrarian) | 1 (1,1) |  | 1 (1,1) |  | 1 (1,1) |  |
| Pastoralist | 0.97 (0.57, 1.67) | 0.920 | 0.56 (0.38, 0.83) | 0.004*** | 0.52 (0.40, 0.68) | 0.000*** |
| City | 38.23 (22.00, 66.42) | 0.000*** | 15.16 (10.18, 22.55) | 0.000*** | 6.60 (4.96, 8.77) | 0.000*** |

*sig. at 10% level; **sig. at 5% level; ***sig. at 1% level; ^1^Orthodox, Catholic, Protestant ^2^Traditional, and other unspecified; COR: (Crude)Unadjusted odds ratios; ref: reference category
