# Supplementary material for: Social Determinants of Antenatal Care Service Use in Ethiopia: Changes Over a 15-Year Span
Source: Front Public Health. 2019 Jun 25;7:161. doi: 10.3389/fpubh.2019.00161 (PMC6603173; doi:10.3389/fpubh.2019.00161)
Supplement: Supplementary file 2 [file Table_2.docx]

**Additional Table 2**. Changes in the number of ANC visits attended according to selected determinants presented as adjusted incident rates ratios (aIRR) in the 2011 and 2016 surveys relative to the 2005 Ethiopian Demographic Health Surveys.

| **Survey periods** | **2011** | | **2016** | |
| --- | --- | --- | --- | --- |
| Covariates | aIRR (95% CI) | *P*-value | aIRR (95% CI) | *P*-value |
| **Overall time effect** (ref: 2005) | 1.51 (1.38, 1.64) | < 0.01 | 2.84 (2.62, 3.09) | < 0.01 |
| **Individual level SD** |  |  |  |  |
| **Age when giving last birth** (years) |  |  |  |  |
| < 20 | 1.43 (1.17, 1.76) | < 0.01 | 2.70 (2.12, 3.30) | < 0.01 |
| 20 – 34 | 1.52 (1.38, 1.68) | < 0.01 | 2.92 (2.64, 3.21) | < 0.01 |
| 35 – 49 | 1.50 (1.30, 1.73) | < 0.01 | 2.74 (2.39, 3.14) | < 0.01 |
| **Order of the last birth** |  |  |  |  |
| First | 1.09 (0.91, 1.30) | 0.36 | 1.92 (1.62, 2.27) | < 0.01 |
| Second or third | 1.69 (1.52, 1.87) | < 0.01 | 3.12 (2.81, 3.46) | < 0.01 |
| Fourth or higher | 1.48 (1.30, 1.69) | < 0.01 | 2.99 (2.63, 3.40) | < 0.01 |
| **Religion** |  |  |  |  |
| Christianity^1^ | 1.62 (1.46, 1.80) | < 0.01 | 3.01 (2.72, 3.33) | < 0.01 |
| Islam | 1.26 (1.09, 1.46) | < 0.01 | 2.43 (2.11, 2.80) | < 0.01 |
| Others^2^ | 2.80 (1.57, 5.02) | <0.01 | 7.15 (3.95, 12.95) | < 0.01 |
| **Women’s education level** |  |  |  |  |
| No education | 1.60 (1.46, 1.77) | < 0.01 | 3.08 (2.81, 3.39) | < 0.01 |
| Primary | 1.27 (1.09, 1.49) | < 0.01 | 2.47 (2.12, 2.88) | < 0.01 |
| Secondary and above | 1.32 (0.88, 2.00) | 0.18 | 1.59 (1.14, 2.23) | 0.01 |
| **Woman’s employment status** |  |  |  |  |
| Not employed | 1.39 (1.25, 1.54) | < 0.01 | 2.79 (2.53, 3.08) | < 0.01 |
| Employed | 1.68 (1.49, 1.91) | < 0.01 | 3.01 (2.65, 3.41) | < 0.01 |
| **Partner’s education level** |  |  |  |  |
| No education | 1.41 (1.26, 1.57) | < 0.01 | 2.81 (2.54, 3.12) | < 0.01 |
| Primary | 1.67 (1.48, 1.90) | < 0.01 | 3.19 (2.82, 3.61) | < 0.01 |
| Secondary and above | 1.62 (1.27, 2.07) | < 0.01 | 2.23 (1.81, 2.73) | < 0.01 |
| **Partner’s employment status** |  |  |  |  |
| Not employed | 1.41 (0.90, 2.19) | 0.13 | 2.01 (1.49, 2.71) | < 0.01 |
| Employed | 1.52 (1.39, 1.65) | < 0.01 | 2.91 (2.68, 3.17) | < 0.01 |
| **In a polygamous relationship** |  |  |  |  |
| No | 1.53 (1.40, 1.67) | < 0.01 | 2.83 (2.59, 3.08) | < 0.01 |
| Yes | 1.36 (1.10, 1.68) | 0.01 | 3.02 (2.46, 3.71) | < 0.01 |
| **Household wealth index** |  |  |  |  |
| Low | 1.68 (1.48, 1.89) | < 0.01 | 3.44 (3.05, 3.88) | < 0.01 |
| Middle | 1.48 (1.27, 1.72) | < 0.01 | 3.14 (2.72, 3.64) | < 0.01 |
| High | 1.37 (1.21, 1.57) | < 0.01 | 2.17 (1.92, 2.46) | < 0.01 |
| **Exposure to Media** |  |  |  |  |
| No mass media exposure | 1.36 (1.22, 1.50) | < 0.01 | 3.00 (2.72, 3.30) | < 0.01 |
| Exposed to either radio or TV | 1.61 (1.40, 1.86) | < 0.01 | 2.84 (2.41, 3.35) | < 0.01 |
| Exposed to both radio and TV | 1.60 (1.30, 1.96) | < 0.01 | 2.23 (1.82, 2.75) | < 0.01 |
| **Sex of household head** |  |  |  |  |
| Male headed | 1.54 (1.41, 1.68) | < 0.01 | 2.89 (2.65, 3.15) | < 0.01 |
| Female headed | 1.23 (0.98, 1.55) | 0.07 | 2.44 (1.96, 3.05) | < 0.01 |
| **Self-reported empowerment of women** |  |  |  |  |
| Not involved at all in decision making | 0.83 (0.67, 1.02) | 0.08 | 2.01 (1.65, 2.44) | < 0.01 |
| Involved in one | 1.86 (1.56, 2.22) | < 0.01 | 3.99 (3.27, 4.85) | < 0.01 |
| Involved in two | 1.43 (1.23, 1.66) | < 0.01 | 2.99 (2.55, 3.51) | < 0.01 |
| Involved in at least three | 1.65 (1.48, 1.84) | < 0.01 | 2.84 (2.56, 3.15) | < 0.01 |
| **Community level SD** |  |  |  |  |
| **Area of Residence** |  |  |  |  |
| Urban | 1.27 (0.96, 1.69) | 0.10 | 1.48 (1.12, 1.95) | 0.01 |
| Rural | 1.52 (1.39, 1.66) | < 0.01 | 3.09 (2.83, 3.38) | < 0.01 |
| **Contextual Region** |  |  |  |  |
| Agrarian | 1.55 (1.42, 1.70) | < 0.01 | 3.01 (2.75, 3.29) | < 0.01 |
| Pastoralist | 1.23 (0.91, 1.67) | 0.17 | 2.22 (1.67, 2.96) | < 0.01 |
| City | 1.00 (0.58, 1.72) | 0.99 | 1.06 (0.61, 1.84) | 0.84 |

^1^Orthodox, Catholic, Protestant ^2^Traditional, and other unspecified; aIRR: adjusted incidence rate ratios; ref: reference category

**Note**: a: adjusted for: time effect, age at last birth, order of lastbirth, religion, place of residence, region, women education, women employment, husband education, husband employment, polygams relation, wealth, media, sex of household head, empowerment.
