# Supplementary material for: Social Determinants of Antenatal Care Service Use in Ethiopia: Changes Over a 15-Year Span
Source: Front Public Health. 2019 Jun 25;7:161. doi: 10.3389/fpubh.2019.00161 (PMC6603173; doi:10.3389/fpubh.2019.00161)
Supplement: Supplementary file 3 [file Table_3.docx]

**Additional Table 3**. Multiple multilevel logistic regression model showing social determinants associated with having at least four ANC visits by survey year-2005, 2011, and 2016 Ethiopia DHS

| Survey periods | 2005 | | 2011 | | 2016 | |
| --- | --- | --- | --- | --- | --- | --- |
| Covariates | AOR (95% CI) | *P*-value | AOR (95% CI) | *P*-value | AOR (95% CI) | *P*-value |
| **Individual level SD** |  |  |  |  |  |  |
| **Mother’s age at last birth (ref: < 20 years)** |  |  |  |  |  |  |
| 20 – 34 | 1.17 (0.83, 1.64) | 0.38 | 2.15 (1.51, 3.05) | < 0.01 | 1.99 (1.48, 2.68) | < 0.01 |
| 35 – 49 | 1.41 (0.94, 2.11) | 0.10 | 2.90 (1.94, 4.32) | < 0.01 | 2.23 (1.58, 3.17) | < 0.01 |
| **Birth order of the last birth (ref: Birth order, 1)** |  |  |  |  |  |  |
| Birth order, 2 - 3 | 0.48 (0.36, 0.65) | < 0.01 | 0.69 (0.53, 0.89) | < 0.01 | 1.02 (0.82, 1.26) | 0.88 |
| Birth order, 4+ | 0.37 (0.26, 0.54) | < 0.01 | 0.58 (0.42, 0.80) | < 0.01 | 0.75 (0.57, 1.00) | 0.05 |
| **Religion (ref:Christianity^1^)** |  |  |  |  |  |  |
| Islam | 0.93 (0.72, 1.20) | 0.58 | 0.73 (0.59, 0.91) | < 0.01 | 0.82 (0.68, 0.99) | 0.04 |
| Others^2^ | 0.71 (0.24, 2.09) | 0.53 | 0.82 (0.34, 1.96) | 0.65 | 0.76 (0.35, 1.63) | 0.48 |
| **Women’s education level (ref: No education)** |  |  |  |  |  |  |
| Primary | 1.67 (1.25, 2.22) | < 0.01 | 1.55 (1.25, 1.92) | < 0.01 | 1.27 (1.06, 1.53) | 0.01 |
| Secondary and above | 3.91 (2.55, 6.02) | < 0.01 | 2.37 (1.57, 3.58) | < 0.01 | 1.17 (0.88, 1.55) | 0.29 |
| **Woman’s employment status (ref: Not employed)** |  |  |  |  |  |  |
| Employed | 1.22 (0.95, 1.56) | 0.11 | 1.29 (1.06, 1.57) | 0.01 | 1.00 (0.85, 1.18) | 0.98 |
| **Partner’s education level (ref: No education)** |  |  |  |  |  |  |
| Primary | 1.12 (0.85, 1.48) | 0.42 | 1.51 (1.22, 1.87) | < 0.01 | 1.08 (0.90, 1.30) | 0.41 |
| Secondary and above | 2.47 (1.81, 3.38) | < 0.01 | 1.83 (1.34, 2.50) | < 0.01 | 0.97 (0.76, 1.23) | 0.79 |
| **Partner’s employment status (ref: Not employed)** |  |  |  |  |  |  |
| Employed | 0.74 (0.47, 1.17) | 0.20 | 0.96 (0.59, 1.56) | 0.88 | 1.06 (0.86, 1.30) | 0.59 |
| **In a polygamous relationship (ref: No)** |  |  |  |  |  |  |
| Yes | 1.02 (0.71, 1.47) | 0.91 | 0.84 (0.61, 1.15) | 0.28 | 0.85 (0.66, 1.10) | 0.21 |
| **Household wealth index (ref: Low)** |  |  |  |  |  |  |
| Middle | 1.43 (0.97, 2.11) | 0.07 | 1.46 (1.08, 1.98) | 0.01 | 1.37 (1.09, 1.72) | 0.01 |
| High | 3.21 (2.38, 4.34) | < 0.01 | 2.27 (1.78, 2.88) | < 0.01 | 1.33 (1.08, 1.62) | 0.01 |
| **Exposure to Media(ref: No mass media exposure)** |  |  |  |  |  |  |
| Exposed to either radio or TV | 1.38 (1.06, 1.80) | 0.02 | 1.29 (1.00, 1.66) | 0.05 | 1.32 (1.04, 1.68) | 0.02 |
| Exposed to both radio and TV | 1.96 (1.39, 2.76) | < 0.01 | 1.98 (1.57, 2.50) | < 0.01 | 1.11 (0.91, 1.36) | 0.30 |
| **Sex of household head(ref: Male headed)** |  |  |  |  |  |  |
| Female headed | 1.75 (1.24, 2.48) | < 0.01 | 0.80 (0.61, 1.06) | 0.12 | 0.99 (0.80, 1.23) | 0.96 |
| **Women’s empowerment (ref: Not involved at all)** |  |  |  |  |  |  |
| Involved in a single major decision | 0.86 (0.55, 1.34) | 0.52 | 1.45 (0.95, 2.20) | 0.09 | 1.58 (1.09, 2.29) | 0.02 |
| Involved in two major decisions | 1.07 (0.71, 1.61) | 0.76 | 1.45 (0.98, 2.15) | 0.06 | 1.10 (0.78, 1.55) | 0.58 |
| Involved in at least three major decisions | 1.07 (0.73, 1.57) | 0.74 | 1.91 (1.34, 2.72) | < 0.01 | 1.07 (0.81, 1.42) | 0.62 |
| **Community level SD** |  |  |  |  |  |  |
| **Area of Residence(ref: Urban )** |  |  |  |  |  |  |
| Rural | 0.19 (0.14, 0.26) | < 0.01 | 0.35 (0.27, 0.45) | < 0.01 | 0.75 (0.59, 0.96) | 0.02 |
| **Region (ref:Agrarian)** |  |  |  |  |  |  |
| Pastoralist | 1.58 (1.17, 2.13) | < 0.01 | 0.85 (0.66, 1.10) | 0.22 | 0.74 (0.59, 0.92) | 0.01 |
| City | 3.19 (2.23, 4.57) | < 0.01 | 2.46 (1.84, 3.28) | < 0.01 | 1.82 (1.39, 2.38) | < 0.01 |

*sig. at 10% level; **sig. at 5% level; ***sig. at 1% level; ^1^Orthodox, Catholic, Protestant ^2^Traditional, and other unspecified

AOR: adjusted odds ratios; ref: reference category

Notes: adjusted for: mothage, birthorder, religion, resid.place, region, mothedu, moth.employ, husedu, hus.employ, polygams, wealth, media, empowerment.
